# Editorial: Synthetic peptide vaccine platforms targeting tumor-specific antigens: advances and challenges

**DOI:** 10.3389/fphar.2024.1363282

**Published:** 2024-02-23

**Authors:** Reid M. Rubsamen, Andrew E. Sloan

**Affiliations:** ^1^ School of Medicine, Case Western Reserve University, Cleveland, OH, United States; ^2^ University Hospitals Cleveland Medical Center, Cleveland, OH, United States; ^3^ Piedmont Healthcare, Atlanta, GA, United States

**Keywords:** peptide vaccine, synthetic vaccines, tumor specific antigens, toll like receptor (TLR), MHC binding affinity prediction, TCR sequencing, immunogenicity, ELISpot assay

Contributors to our Research Topic have explored issues related to both the discovery and therapeutic aspects of peptide vaccine platforms, specifically in relation to identifying potential antigen targets on tumors. This involves two main areas of focus: the discovery of potentially immunogenic tumor peptide antigen targets and strategies to enhance the immunogenicity of these targets.

Contributions range from description of novel computational techniques for identifying potentially immunogenic peptide antigens on tumors to assessing novel combinations of antigens and TLR agonists.

Advancements in tools for predicting peptide binding to Major Histocompatibility Complex (MHC) are noteworthy, including the integration of deep learning AI in HLA/peptide combination binding predictors (Jumper et al., 2021). It is crucial to emphasize that while MHC binding affinity is a valuable screening metric, it alone does not predict immunogenicity: MHC binding is a necessary but not sufficient condition for specific T-cell expansion and to evoke T-cell effector function (Lee et al., 2021). We believe that confirmation through *in-vivo* or *in-vitro* determination of actual TCR MHC/peptide binding characteristics remains an essential part of peptide vaccine design. This confirmation can be achieved indirectly using techniques such as ELISPOT or directly through newer approaches such as TCR sequencing (Ranieri et al., 2021; de Greef Peter et al., 2023).

TLR agonists have also proven to be integral to the latest generation of vaccines. Notably, TLR-4 and TLR-9 agonists have been incorporated into FDA-approved vaccines, significantly improving the immunogenicity of Virus-Like Particle (VLP) and other vaccine designs (Luchner et al., 2021). The use of TLR adjuvants becomes especially significant in the design of synthetic peptide cancer vaccines, considering that exogenously administered short peptides lack the immunogenicity of larger proteins (Jeannin et al., 1993; Khong and Overwijk, 2016).

Addressing this issue, some researchers have opted for single long peptides (SLPs) (Abd-Aziz and Poh, 2022). However, these present challenges as enzyme degradation during antigen processing may cleave SLPs into unintended smaller sequences (Garstka et al., 2015). Even a minor deviation of one amino acid from the predicted immunogenic peptide sequence by vaccine designers may result in the delivered peptide no longer being immunogenic (Gao et al., 2001; Herst et al., 2020).

While mRNA vaccines theoretically enhance the immunogenicity of desired peptide antigen targets by producing peptides within cells via the canonical pathway, recent publications have raised concerns about potential transcription errors inherent to mRNA vaccines (Acevedo-Whitehouse and Bruno, 2023; Gunter et al., 2023; Mulroney et al., 2023). This could lead to situations similar to those observed with SLPs, where delivered peptides deviate from the intended sequence and become non-immunogenic.

Synthetic peptide vaccine platforms offer potential advantages, potentially avoiding the manufacturing complexity and safety issues associated with mRNA vaccines and viral vectors. The tools available for designing peptide vaccines for oncology applications are increasing, holding promise for further developments in the field.
